# Enhanced CH_3_OH selectivity in CO_2_ hydrogenation using Cu-based catalysts generated *via* SOMC from Ga^III^ single-sites†

**DOI:** 10.1039/d0sc00465k

**Published:** 2020-02-26

**Authors:** Erwin Lam, Gina Noh, Ka Wing Chan, Kim Larmier, Dmitry Lebedev, Keith Searles, Patrick Wolf, Olga V. Safonova, Christophe Copéret

**Affiliations:** Department of Chemistry and Applied Biosciences, ETH Zurich Vladimir Prelog Weg 2 CH-8093 Zurich Switzerland ccoperet@ethz.ch; Paul Scherrer Institute CH-5232 Villigen Switzerland

## Abstract

Small and narrowly distributed nanoparticles of copper alloyed with gallium supported on silica containing residual Ga^III^ sites can be obtained *via* surface organometallic chemistry in a two-step process: (i) formation of isolated Ga^III^ surface sites on SiO_2_ and (ii) subsequent grafting of a Cu^I^ precursor, [Cu(O^*t*^Bu)]_4_, followed by a treatment under H_2_ to generate CuGa_*x*_ alloys. This material is highly active and selective for CO_2_ hydrogenation to CH_3_OH. *In situ* X-ray absorption spectroscopy shows that gallium is oxidized under reaction conditions while copper remains as Cu^0^. This CuGa material only stabilizes methoxy surface species while no formate is detected according to *ex situ* infrared and solid-state nuclear magnetic resonance spectroscopy.

## Abstract 2

CuGa*_x_* alloy nanoparticles supported on SiO_2_ can be obtained by surface organometallic chemistry. This catalyst is active and selective for the hydrogenation of CO_2_ to CH_3_OH, related to the formation of an optimal interface between Cu and Ga^III^O*_x_*.

## Graphical abstract

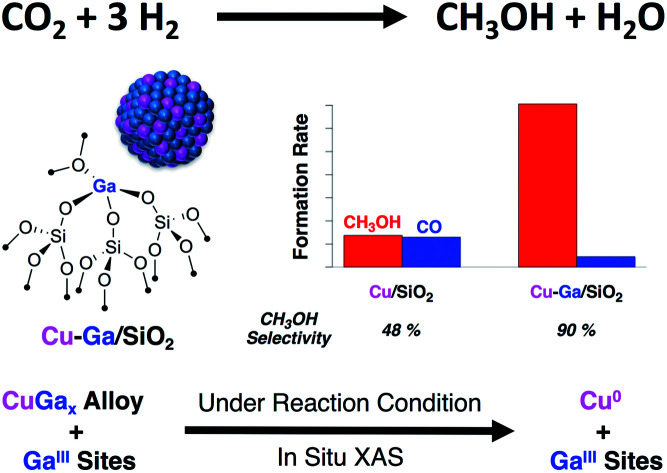

## Introduction

The transformation of CO_2_ into value-added products is a promising strategy to mitigate the further increase of CO_2_ in the earth's atmosphere that is connected to environmental changes.^1–4^ In that context, the hydrogenation of CO_2_ yields CH_3_OH that can be used as a base chemical for the production of fuels or as a fuel itself, thus generating a closed carbon-fuel-cycle, provided that efficient CO_2_ capture and storage, and efficient H_2_ production from renewable energy sources are available.^5^ Major issues associated with the hydrogenation of CO_2_ into CH_3_OH are the selectivity of the process and the long-term stability of catalysts. Regarding selectivity, the hydrogenation of CO_2_ can also yield CO *via*, for instance, the reverse water gas shift (RWGS) reaction or the decomposition of methyl formate, that can be formed *in situ via* secondary reactions.^6,7^ Heterogeneous catalysts that are able to form CH_3_OH with good selectivity and activity mainly consist of copper-based catalysts together with zinc oxide (*e.g.* Cu/ZnO or Cu/ZnO/Al_2_O_3_), gallium oxide (*e.g.* Cu/ZnO/Ga_2_O_3_) or zirconium oxide (Cu/ZrO_2_).^8–23^ In the case of ZrO_2_, it has been shown that the Lewis acidic surface Zr^IV^ sites stabilize reaction intermediates (CO_2_, formates and methoxy) and improve the activity and methanol selectivity. The origin of the promotional effect of zinc or gallium oxide is, however, not fully understood.^24–29^ For instance, the formation of highly active CuZn surface alloys or interfacial sites between Cu and ZnO have both been proposed.^24–29^ The promotional effect of gallium oxide has been far less studied.^27,30^

**Scheme 1:**
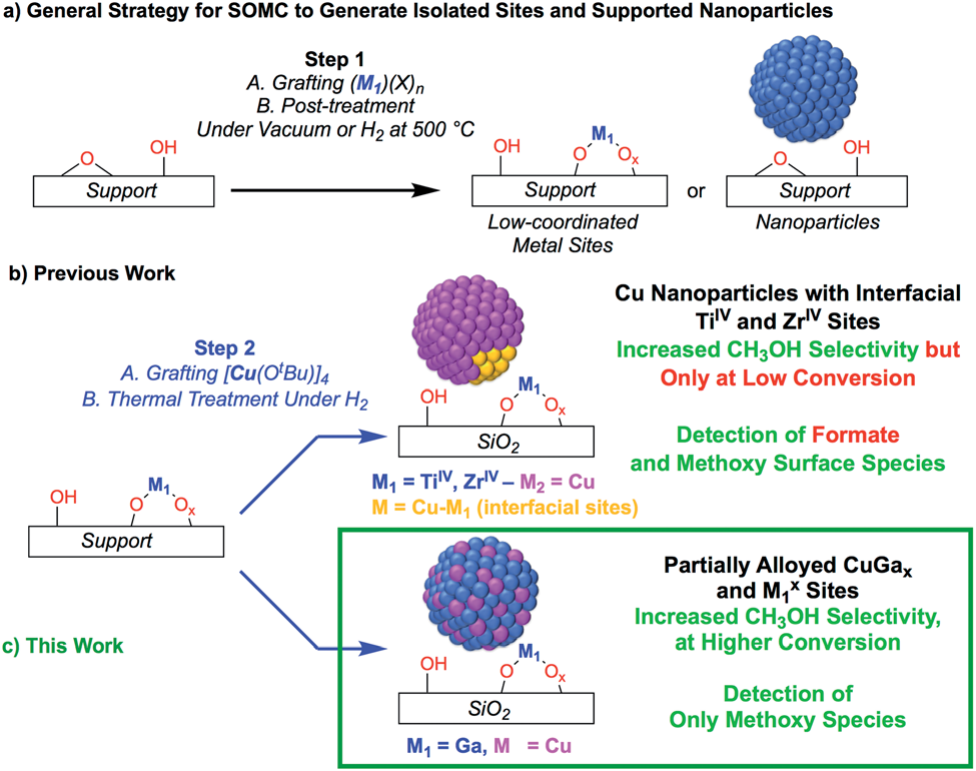
(a) General SOMC strategy to generate isolated sites or supported nanoparticles. (b and c) Utilizing isolated metal site supports generated from SOMC for metal and metal–alloy nanoparticle formation.

Recently, it has been shown that surface organometallic chemistry (SOMC) combined with thermolytic molecular precursors (TMP) constitutes a powerful synthetic strategy to generate supported metal nanoparticles surrounded by well-defined isolated promoter sites that allows investigating the role of interfacial sites *via in situ* spectroscopic methods.^31,32^ This approach can also yield supported alloyed nanoparticles, depending on the selection of metal and promoters (*vide infra*). The SOMC/TMP approach can be summarized as follows: in a first step, the support, SiO_2_, is dehydroxylated at 700 °C to obtain isolated surface silanol (Si–OH) groups (1 OH, nm^−2^) that are used as anchoring groups to graft the TMP.^33^ Post-thermal treatment at high temperature generates isolated low-coordinated metal surface sites, that are free of organic ligands, and restores ≡Si–OH groups (Scheme 1a), onto which a second molecular precursor is grafted. Subsequent post-treatment under reducing conditions (H_2_) generates metal nanoparticles interfacing low-coordinated metal surface sites (Scheme 1b). This approach has allowed the generation of highly active and selective CO_2_ hydrogenation catalysts by supporting Cu nanoparticles on SiO_2_ containing isolated Zr^IV^ and Ti^IV^ sites.^34,35^ These catalysts show high activity and CH_3_OH selectivity, but also suffer from the decrease of CH_3_OH selectivity at high conversion as observed for other CO_2_ hydrogenation catalysts.^34^ The outstanding activity and CH_3_OH selectivity of copper supported on silica containing Ti^IV^ isolated sites is particularly noteworthy, considering that Cu/TiO_2_ performs very poorly in CH_3_OH synthesis by favoring CO formation.^36–39^ This difference of catalyst performance has been ascribed to the site isolation of Ti^IV^ and the use of a non-reducible support, SiO_2_, thus allowing Ti^IV^ to play exclusively the role of a Lewis acid, that stabilizes reaction intermediates at the interface with Cu particles.^35^ Using a similar approach, *i.e.*, the treatment under H_2_ of a grafted platinum(ii) molecular precursor on isolated Ga^III^ sites generates small and narrowly distributed PtGa_*x*_ nanoparticles stabilized by remaining Ga^III^ sites that show high activity, selectivity and stability for propane dehydrogenation.^40^

Here, we reasoned that the SOMC/TMP approach would be a useful tool to investigate the behavior of gallium promoters in CO_2_ hydrogenation catalysts (Scheme 1c). We thus prepared Cu-based nanoparticles by grafting [Cu(O^*t*^Bu)]_4_ on a silica support containing Ga^III^ single-sites, followed by a treatment under H_2_. This approach yields nanoparticles consisting of CuGa_*x*_ alloys that evolved under CO_2_ hydrogenation conditions into Cu^0^ and Ga^III^ according to *in situ* X-ray absorption spectroscopy (XAS). Such **Cu-Ga/SiO2** catalysts display enhanced activity and selectivity at higher conversions in the hydrogenation of CO_2_ into CH_3_OH by comparison to other Cu-based catalysts. These improved performances are attributed to Cu^0^/Ga^III^ interfaces that only stabilize methoxy and not formate intermediates, according to infrared (IR) and solid state nuclear magnetic resonance (NMR) spectroscopy.

## Results and discussion

### Catalyst synthesis and characterization

**Fig. 1:**
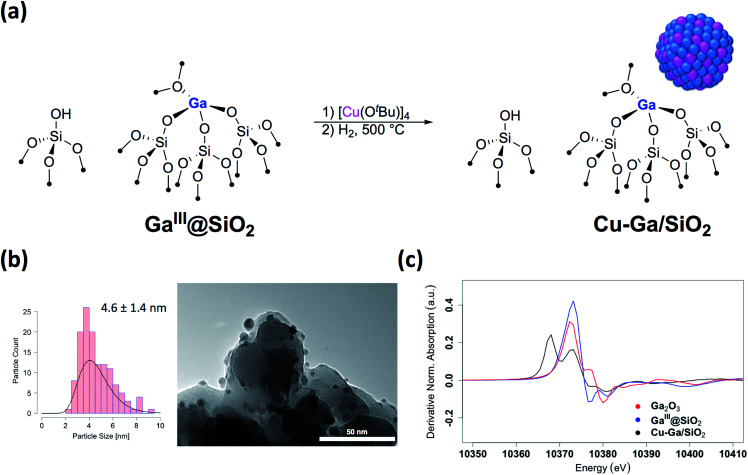
(a) Schematic procedure for grafting of [Cu(O^*t*^Bu)]_4_ on **GaIII@SiO2** followed by reduction under H_2_ at 500 °C. (b) Particle size distribution and TEM images of **Cu-Ga/SiO2**. (c) First derivative of the XANES spectra at the Ga K-edge of **Cu-Ga/SiO2** and reference samples.

We first prepared well-defined Ga^III^ sites on SiO_2_ with *ca.* 1.0 Ga^III^ nm^−2^ by grafting and thermolysis of [Ga(OSi(O^*t*^Bu)_3_)_3_(THF)]:^41,42^**GaIII@SiO2** (ref. 42) (**Mx@SiO2** denoted as isolated M surface sites in its *x* oxidation state) *via* an SOMC/TMP approach and then grafted [Cu(O^*t*^Bu)]_4_ on residual surface silanols present in **GaIII@SiO2**. The IR spectra show the consumption of Si–OH groups (3747 cm^−1^) and the appearance of C–H stretching (2700–3000 cm^−1^) and bending (1300–1500 cm^−1^) bands, consistent with grafting of [Cu(O^*t*^Bu)]_4_*via* protonolysis of the Si–OH group (Fig. S1†). Next, reduction under H_2_ at 500 °C removes all the organics and regenerate the Si–OH groups, as shown by IR spectroscopy, yielding **Cu-Ga/SiO2** (Fig. 1a). Based on inductively coupled plasma optical emission spectroscopy (ICP-OES), a metal loading of 1.61 wt% gallium and 3.88 wt% copper is obtained for **Cu-Ga/SiO2** (corresponding to a 5 : 2 Cu : Ga atomic ratio).

A specific surface area of *ca.* 200 m^2^ g^−1^ – determined by N_2_ physisorption isotherms and Brunauer–Emmett–Teller^43^ (BET) analysis (Table S1†) – is obtained, similar to this of the initial material. Transmission electron microscopy (TEM) studies show the formation of small and narrowly distributed nanoparticles on SiO_2_ (4.6 ± 1.4 nm) (Fig. 1b), which corresponds to Cu–Ga alloy nanoparticles (*vide infra*). These particles are slightly larger than what was found for the corresponding **Cu/SiO2** (2.9 ± 1.3 nm) prepared *via* a similar approach.^34^ Energy-dispersive X-ray (EDX) mapping of as-prepared **Cu-Ga/SiO2**, introduced to the microscope without exposure to air using a vacuum transfer TEM sample holder, shows that gallium is found in the same region as the copper nanoparticles, but it is also dispersed throughout the support (Fig. S2†). In addition, the particle size distribution of **Cu-Ga/SiO2** from TEM of samples with and without exposure to air does not change, indicating the absence of copper and gallium redispersion under oxidizing conditions (Fig. S3†). N_2_O titration at 90 °C, that selectively titrates the surface of Cu nanoparticles, was performed, resulting in around 55 μmol g_cat_^−1^ surface sites for **Cu-Ga/SiO2** (assuming a 1 : 2 stoichiometry between N_2_O and the Cu surface site) (Table S1†), which is similar for what is obtained for **Cu/SiO2** (50 μmol g_cat_^−1^),^34^ despite the larger particle sizes for **Cu-Ga/SiO2**. It is possible that N_2_O reacts with reduced gallium sites leading to a higher N_2_O uptake similar to what is observed with zinc in case of Cu/ZnO/Al_2_O_3_.^24^ Chemisorption experiments using H_2_ at 40 °C indicate around 60 μmol g_cat_^−1^ surface sites (assuming a 1 : 2 stoichiometry between H_2_ and the surface site) for **Cu-Ga/SiO2**, consistent with the number obtained *via* N_2_O titration (Table S1 and Fig. S4†). No crystalline phases are observed by powder X-ray diffraction, as expected from the amorphous nature of the SiO_2_ support and the presence of small metal nanoparticles (Fig. S5†). The presence of residual Lewis acidic gallium sites is supported by pyridine adsorption and IR spectroscopy,^44^ by the presence of ring vibrational bands of pyridine at 1621 cm^−1^, characteristic for Lewis acidic surface sites, likely associated with Ga^III^ sites (Fig. S6†). Upon adsorption of pyridine, it persists even at 500 °C under high-vacuum (10^−5^ mbar) indicating strongly bound pyridine (for details see ESI†). IR spectroscopy (Fig. S7†) of the catalysts upon adsorption of CO (90 mbar) at room temperature shows stretching bands at 2102 cm^−1^ for **Cu-Ga/SiO2** corresponding to CO bonded to the metal nanoparticle which are slightly red-shifted with respect to what is observed for pure **Cu/SiO2** at 2106 cm^−1^.

Further information regarding the oxidation states and structural environment of copper and gallium in **Cu-Ga/SiO2** is obtained by the XAS spectra at the copper and gallium K-edges for the as-prepared catalysts stored under inert conditions (Fig. 1c). The Cu K-edge X-ray absorption near-edge structure (XANES) spectrum of **Cu-Ga/SiO2** shows an edge energy at 8979 eV consistent with reduced Cu^0^ but the near edge features of **Cu-Ga/SiO2** are different from the **Cu/SiO2** or Cu foil spectrum (Fig. S8†). Fitting of the extended X-ray absorption fine structure (EXAFS) shows the presence of gallium (*N* = 4 ± 3 at 2.57 Å) and copper (*N* = 7 ± 2 at 2.54 Å) scattering paths (Table 1 and Fig. S9†), suggesting the formation of a CuGa_*x*_ alloy phase. The XANES spectrum of **Cu-Ga/SiO2** at the gallium K-edge shows an edge energy of 10 368 eV, which is 5 eV lower than the edge energy of **GaIII@SiO2** or Ga_2_O_3_, as reference samples (Fig. 1c and S10†). The shift in the edge energy indicates the presence of reduced gallium species, while the feature observed in **GaIII@SiO2** or Ga_2_O_3_ at 10 373 eV is still present, albeit lower in intensity, in **Cu-Ga/SiO2** as shown in the first derivative of the XANES spectrum (Fig. 1c). Further analysis and fitting of the EXAFS (Table 1) at the Ga K-edge reveals the presence of Cu neighbors (*N* = 8 ± 2 at 2.57 Å) consistent with the EXAFS fitting result at the Cu K-edge with part of the gallium forming a metal alloy phase with copper. In addition, there are oxygen neighbors (*N* = 2 ± 1 at 1.81 Å), which are attributed to remaining isolated Ga^III^ sites (Fig. S11†), consistent with pyridine adsorption and IR spectroscopy (*vide supra*). Considering the number of oxygen neighbors (*N* = 4) found in **GaIII@SiO2**,^42^ we estimated that approx. 50% of Ga is in the form of Ga^III^ sites in **Cu-Ga/SiO2**. This indicates formation of CuGa_*x*_ alloy with *x* ≈ 0.2 based on respective copper (3.88 wt%) and gallium (1.61 wt%) loadings and taking into account that *ca.* 50% of gallium sites are present in its reduced form.

**Table 1:** EXAFS fits parameters of **Cu-Ga/SiO2** at the Cu and Ga K-edges

Edge	Neighbor, *N*^a^	*r* [Å]^b^	*σ* ^2^ [Å^2^]^c^
Cu K-edge	Ga, 4 ± 3	2.57 ± 0.01	0.012 ± 0.002
	Cu, 7 ± 2	2.54 ± 0.02	0.0100 ± 0.0009
Ga K-edge	O, 2 ± 1	1.81 ± 0.03	0.012 ± 0.009
	Cu, 8 ± 2	2.57 ± 0.01	0.012 ± 0.002

a Number of specified neighbors.

b Distance to corresponding neighbor.

c Debye–Waller factor.

Overall, the XAS spectra show that reduction of the samples after Cu grafting (500 °C under H_2_) leads to a partial reduction of Ga^III^ with the formation of CuGa_*x*_ alloys along with remaining Ga^III^ sites. These finding contrast with what was observed for **Cu-Ti/SiO2** (ref. 35) and **Cu-Zr/SiO2** (ref. 34) prepared in a similar fashion from Ti^IV^ and Zr^IV^ single-sites that remained isolated upon Cu nanoparticle formation.

### Catalytic performance in CO_2_ hydrogenation

**Fig. 2:**
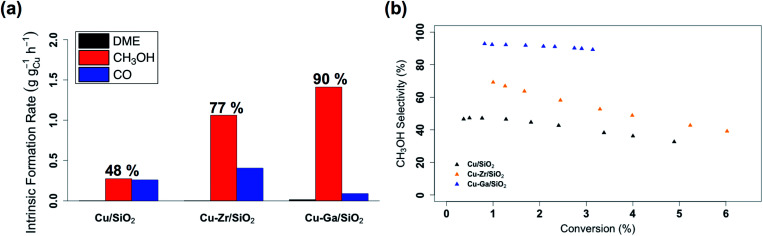
(a) Intrinsic formation rates for CO, CH_3_OH and DME and (b) overall CH_3_OH selectivity *vs.* conversion for **Cu/SiO2**, **Cu-Zr/SiO2** and **Cu-Ga/SiO2**.

The catalytic performance of **Cu-Ga/SiO2** in CO_2_ hydrogenation was evaluated at 230 °C under a total pressure of 25 bar and 3 : 1 H_2_ : CO_2_. Following exposure to air, the material is reduced at 300 °C under H_2_ in the reactor prior to catalysis. The effect of contact time on the catalytic activity/selectivity by varying the gas flowrate and the intrinsic formation rates extrapolated to zero contact time are evaluated and compared with **Cu/SiO2** and **Cu-Zr/SiO2** benchmark materials (Fig. S12 and S13†). Catalytic tests are carried out at conversions below 7%, which are far from thermodynamic equilibrium (15% with a CH_3_OH selectivity of 43%)^6^ under the given reaction condition. The intrinsic formation rate for CH_3_OH is 1.3 g h^−1^ g_Cu_^−1^ for **Cu-Ga/SiO2** that is 4 times higher than **Cu/SiO2** and also slightly higher than **Cu-Zr/SiO2** (Fig. 2a). Note that the product formation rates on the support itself (**GaIII@SiO2**) are below detection limits. The intrinsic CO formation rate of **Cu-Ga/SiO2** (0.1 g h^−1^ g_Cu_^−1^) is 3 times lower as compared to **Cu/SiO2** or **Cu-Zr/SiO2** (0.3 g h^−1^ g_Cu_^−1^), making **Cu-Ga/SiO2** a better catalysts with an improved selectivity to CH_3_OH products of 93% (CH_3_OH/DME = 30) with only 7% of CO. The formation of DME likely arise from the subsequent dehydration of CH_3_OH, indicating a significant Lewis acidity for this support (*vide supra*).^45^ Remarkably, **Cu-Ga/SiO2** shows a higher CH_3_OH selectivity than unpromoted **Cu/SiO2** (48%) or even **Cu-Zr/SiO2** (77%).^34^ At longer contact times (Fig. S14†), both CH_3_OH and CO formation rates decrease (with a slightly larger decrease for CH_3_OH formation rates (Fig. S14†)), suggesting product inhibition for both processes for **Cu-Ga/SiO2**. The decrease of activity with contact time is a key limiting factor for a high productivity of CH_3_OH for the **Cu-Ga/SiO2** catalyst. The product inhibition is likely associated with the blocking of Lewis acidic Ga^III^ sites by H_2_O/CH_3_OH that in turn reduces CH_3_OH and CO formation rates. Note that in case of **Cu/SiO2**, where no Lewis acidic (interfacial) sites are present to assist the conversion of CO_2_, CH_3_OH and CO formation rates remain independent of contact time. Product inhibition was also observed for the related **Cu-Ti/SiO2** and **Cu-Zr/SiO2** catalysts. However, the major difference between **Cu-Ti/SiO2** or **Cu-Zr/SiO2** and **Cu-Ga/SiO2** is that in the former cases, the contact time affects the CH_3_OH formation rates more than CO formation rates, leading to a decrease of CH_3_OH selectivity. This dramatic decrease of selectivity is not observed for **Cu-Ga/SiO2**, also indicating that CO likely forms *via* different mechanisms. A high selectivity toward CH_3_OH is maintained for **Cu-Ga/SiO2** (>89%; taking into account DME that is initially formed from CH_3_OH) at *ca.* 3% conversions *vs.* 71% and 60% selectivity for **Cu-Ti/SiO2** and **Cu-Zr/SiO2**, respectively at the same conversion (Fig. 2b and S15†). After 30 hours of reaction, **Cu-Ga/SiO2** deactivates for both CH_3_OH and CO formation by 75% and 80%, respectively. Analysis of the spent catalyst shows a similar particle size distribution by TEM for **Cu-Ga/SiO2** of 4.9 ± 1.6 nm compared to the fresh catalyst (Fig. S16†). The absence of any crystalline phases by powder X-ray diffraction (Fig. S3†) further indicates the absence of significant sintering throughout the catalytic testing. The deactivation of the catalyst could originate from a slightly decreased amount of accessible metal sites as shown by N_2_O titration of the fresh/spent catalyst (Table S1†).

### *In situ* X-ray absorption spectroscopy

**Fig. 3:**
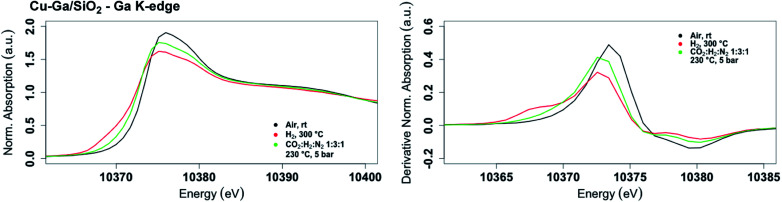
*In situ* XANES spectrum (left) and its first derivative (right) at the gallium K-edge for **Cu-Ga/SiO2** under air at room temperature, reduced at 300 °C under H_2_ and under reaction conditions at 5 bar with CO_2_ : H_2_ : N_2_ (1 : 3 : 1) at 230 °C.

The role of gallium, especially the effect of metal alloy formation and its consequence in promoting the selective formation of CH_3_OH, were further investigated by *in situ* XAS at the copper K-edge and the gallium K-edges for **Cu-Ga/SiO2** (Fig. 3). The X-ray absorption spectra are first recorded after oxidation of the catalyst in air, followed by reduction at 300 °C under H_2_. The reduced catalyst was then cooled down to 230 °C and the reaction gas consisting of CO_2_ and H_2_ (1 : 3) was introduced at 1 bar and then pressurized to 5 bar.

**Fig. 4:**
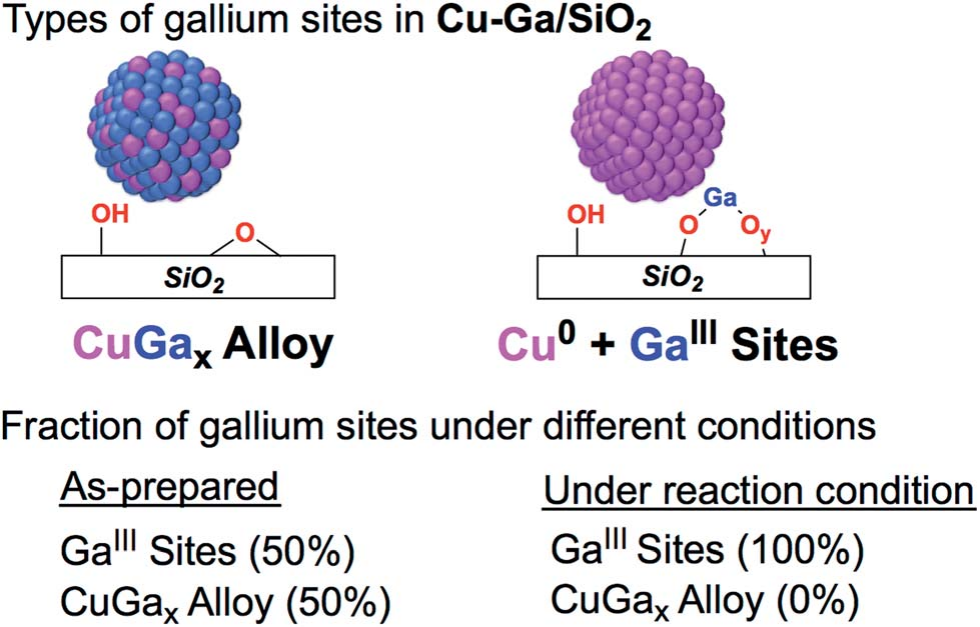
Fraction of gallium sites on **Cu-Ga/SiO2** of the as prepared catalyst and under reaction condition according XAS.

The XANES spectrum of **Cu-Ga/SiO2** at the gallium K-edge after reduction under H_2_ shows a decreased white line intensity and the appearance of a feature at lower energy (10 368 eV) indicative of reduced gallium sites.^42^ The XANES spectrum has a lower intensity of the feature at that energy (10 368 eV) compared to the as-prepared catalyst, indicating that the gallium sites are more difficult to reduce following exposure to air. The feature toward lower energy associated with reduced gallium sites only appears when copper is present and is not observed for **GaIII@SiO2** treated under H_2_ (Fig. S17†). Changes in **GaIII@SiO2** during *in situ* XAS is only due to changes in the oxygen coordination number upon heating at high temperature most likely due to removal of water (due to exposure to air) according to EXAFS fitting (Table S2 and Fig. S18–S20†). The absence of reduced gallium sites for the material without copper (**GaIII@SiO2**) suggests that copper, most likely in close interaction with gallium, is necessary to reduce Ga^III^ to Ga^0^. Under reaction conditions at 5 bar (1 : 3 ratio of CO_2_ : H_2_), the white line intensity increases and the feature toward lower energy disappears, indicating full oxidation of gallium sites (Fig. 4).

Copper is fully reduced upon reaction with H_2_ at 300 °C and under reaction condition according to XAS (Fig. S21†). Furthermore, Lewis acidic surface sites are present in the catalysts after exposure to air followed by reduction as shown by pyridine adsorption and IR spectroscopy (Fig. S22†). This data suggests that the Lewis acidic sites from Ga^III^ would be responsible for the promotion of CH_3_OH synthesis, similarly to what is observed for Cu/ZrO_2_ or related **Cu-Zr/SiO2**.^17,34^

### *Ex situ* solid state NMR spectroscopy

In order to determine possible intermediates on gallium under reaction conditions, ^1^H–^13^C HETCOR spectra of **Cu-Ga/SiO2** (reduced after exposure to air) are recorded after reacting the catalyst with 5 bar of ^1^H_2_ : ^13^CO_2_ (3 : 1) for 12 hours at 230 °C followed by evacuating the gas phase under high vacuum (10^−5^ mbar) at room temperature. The NMR spectra of **Cu-Ga/SiO2** shows a cross-peak at around 3/50 ppm (^1^H/^13^C), which is indicative of methoxy species (Fig. S23†) and the presence of dimethyl ether as evidenced by the additional cross-peak at 3/60 ppm (^1^H/^13^C) (Fig. S23†), consistent with the observed formation of dimethyl ether during catalysis. Notably, no formate species on Lewis acidic gallium sites (present in the case of Cu/ZrO_2_, **Cu-Zr/SiO2**, **Cu-Ti/SiO2** or Cu/Al_2_O_3_ systems)^6,17,34,35^ are observed, indicating that Ga^III^ Lewis acidic sites possibly favor the subsequent hydrogenation of formate into methoxy species and/or increase the thermodynamic stability of methoxy in comparison to formate species. This is also confirmed by the IR spectra of the *ex situ***Cu-Ga/SiO2** sample after reacting with ^1^H_2_/^13^CO_2_ (Fig. S24†) showing the ^13^C–H stretches at around 2954 and 2855 cm^−1^ indicating the presence of methoxy, while no band associated with formate species are observed. While formate species are also likely formed as reaction intermediates on **Cu-Ga/SiO2** under reaction condition, the lower stability of formate compared to methoxy would be consistent with (and explains) the higher CH_3_OH selectivity of this material in contrasts to other systems. Indeed, stable formate species have been shown to be able to generate methyl formate that readily decomposes into CO.^6^ Further work is needed to investigate the formation (or not) of formate species as key intermediate in this **Cu-Ga/SiO2** system.

## Conclusions

The use of SOMC was explored in order to understand the promotional effect of gallium in Cu-based CO_2_ hydrogenation catalysts, starting from well-defined silica-supported Ga^III^ sites as an initial support. This approach generates small and narrowly distributed silica-supported CuGa_*x*_ nanoparticles along with residual Ga^III^ Lewis acidic sites. This is in contrast to previously studied well-defined isolated Zr^IV^ and Ti^IV^ sites on SiO_2_ that yield Cu nanoparticles surrounded with isolated metal interfacial sites.^34,35^ These materials are readily oxidized to generate the corresponding CuO and Ga^III^ sites upon exposure to air, but can be partially reduced back to CuGa_*x*_ alloys under H_2_. These CuGa_*x*_ systems display improved catalytic performances in the hydrogenation of CO_2_, allowing the increase in the overall CH_3_OH (CH_3_OH + DME) selectivity (up to *ca.* 90%) at higher conversion (3%) by comparison with the benchmark catalysts, **Cu-Zr/SiO2** and **Cu-Ti/SiO2**. Under reaction conditions, the silica-supported CuGa_*x*_ de-alloys yielding Cu nanoparticles and Ga^III^ sites indicating that the increased activity and selectivity is likely due to an increased interfacial area between Cu^0^ and Ga^III^O_*x*_ that would promote CH_3_OH formation. In fact, methoxy surface species are the only observed intermediates according to *ex situ* solid state NMR or IR. This study overall shows the subtle difference between promoters; it opens new ways to tailor CH_3_OH selective catalysts. We are currently exploring other promoters to understand their role and to design improved CO_2_ hydrogenation catalysts *via* a more rational design.

## Conflicts of interest

There are no conflicts to declare.

## Supplementary Material

SC-011-D0SC00465K-s001
