# Supplementary material for: Enhanced CH3OH selectivity in CO2 hydrogenation using Cu-based catalysts generated via SOMC from GaIII single-sites
Source: Chem Sci. 2020 Feb 26;11(29):7593–8. doi: 10.1039/d0sc00465k (PMC8159433; doi:10.1039/d0sc00465k)
Supplement: SC-011-D0SC00465K-s001 [file SC-011-D0SC00465K-s001.pdf]

Electronic Supplementary Material (ESI) for Chemical Science.
This journal is © The Royal Society of Chemistry 2020

Supporting Information

Enhanced CH_3OH Selectivity in CO_2 Hydrogenation using Cu-based Catalysts Generated via SOMC from Ga^{III} Single-Sites.

Erwin Lam[a], Gina Noh[a], Ka Wing Chan[a], Kim Larmier[a], Dmitry Lebedev[a], Keith Searles[a], Patrick Wolf[a], Olga V. Safonova[b], Christophe Copéret*[,a]

[a]Department of Chemistry and Applied Biosciences, ETH Zurich, Vladimir Prelog Weg 1-5, CH-8093 Zurich, Switzerland

[b]Paul Scherrer Institute, CH-5232 Villigen, Switzerland

EXPERIMENTAL SECTION

General. SiC (FisherEU), Ga_2O_3 (ABCR), and CH_3OH (Acros Chemicals) were used as received. Toluene was purified over two solvent purification alumina columns (MBraun) and degassed prior to use. H_2 for catalyst preparation was purified over activated R3-11 BASF catalyst and activated 4 Å molecular sieves. Unless otherwise specified all preparations of catalysts were carried out under argon using standard Schlenk techniques and gloveboxes. $[Cu(O^tBu)]_4$[1], Ga^{III}@SiO_2[2] and Cu/SiO_2[3] were synthesized according to literature procedures and stored in an argon filled glovebox.

Synthesis of Cu-Ga/SiO₂: A solution of $[Cu(O^tBu)]_4$ (110 mg, 0.20 mmol) in 20 mL of toluene was added to 1 g of Ga^{III}@SiO_2 wetted with toluene. The suspension was stirred for 4 hours, washed three times with toluene (5 mL) and dried at 10^{-5} mbar for 1 hour. The solid was then reduced under H_2 at 500 °C for 5 hours (100 °C h^{-1}) cooled down to room temperature under H_2, evacuated under high vacuum (10^{-5} mbar) and stored in an argon filled glovebox.

Material characterization. Elemental analyses of all materials were performed by the Mikroanalytisches Labor Pascher, Remagen, Germany. Powder X-ray diffraction (pXRD) patterns were recorded on a PANalytical X'Pert PRO-MPD diffractometer at a voltage of 40 kV and a current of 40 mA by applying Cu-Kα radiation (γ=1.54060 Å). Catalyst morphology was obtained by transmission electron microscopy (TEM) on a Hitachi HT7700 microscope and high-angle annular dark field scanning transmission electron microscopy (HAADF-STEM) and energy dispersive X-ray (EDX) mapping on a Talos F200 X microscope without exposing the samples to air within the facilities of ScopeM at ETH Zurich. For the determination of the particle size distribution, >100 individual particles were considered, and the mean particle size and standard deviation are given according to a lognormal distribution function. Fourier-Transform Infrared (FTIR) spectroscopy experiments were performed on self-supporting wafers using a Bruker Alpha FT-IR spectrometer in transmission mode (24 scans, 4 cm^{-1} resolution) under exclusion of air. The specific surface area of the catalysts was measured from a N_2 physisorption isotherm recorded at 77 K on a BEL JAPAN BELSORP-mini II apparatus. The samples were degassed at 300 °C under vacuum (10^{-3} mbar) for 3 hours prior to measurement. The data was analyzed by the BET method with a p/p^0 range between 0.1 and 0.3. H_2 chemisorption isotherms were obtained using a BELSORP-max apparatus on the reduced samples at 40 °C and fitted according to a Langmuir isotherm (eq. S1),

$$Q_{H_2} = \frac{\sqrt{K_{H_2}\frac{P_{H_2,eq}}{P^0}}}{1+\sqrt{K_{H_2}\frac{P_{H_2,eq}}{P^0}}} Q_{H_2,max} \qquad \text{(S1)}$$

Where $P_{H_2,eq}$ is the equilibrium hydrogen pressure, Q_{H_2} the hydrogen uptake (μmol g_{cat}^{-1}), $Q_{H_2,max}$ the saturation uptake of H_2 and K_{H_2} the thermodynamic constant for the dissociative hydrogen chemisorption.

Copper surface area was determined by N_2O titration. In a typical experiment 30-50 mg of sample were weight into a U-shape quartz tube and connected to the instrument (BEL Japan, INC., BELCAT-B). Prior to analysis, the samples were pretreated under a flow of 50% H_2/He at 300°C for 2h, after which 25-30 successive pulses of the titration gas mixture (1% N_2O in He) were introduced by a calibrated injection valve (2.77 μL_{N_2O} (STP) per pulse). The amount of N_2O consumed is determined by monitoring the amounts of N_2O and N_2 in the exhaust with a thermal conductivity detector. The quantity of surface metal sites $M_{(s)}$ is then determined considering the titration equation[4]:

$$2\,M_{(S)} + N_2O = M_{(S)2}O + N_2 \qquad n_{N_2O} = 2\,n_{M(S)}$$

Pyridine adsorption experiments are performed on a self-supporting pellet of the Cu-based catalyst and monitored by infrared spectroscopy. After exposure of pyridine in the gas phase, the pellet was subsequently placed under high vacuum (10^{-5} mbar) at room temperature (rt), 100 °C, 200 °C, 300 °C, 400 °C and 500 °C (300 °C/h) for 15 minutes prior to measurement of the IR spectrum. The reduction of air

exposed samples was performed at 300 °C (300 °C/h) in the presence of 1 bar H_2 for 1 hour and evacuated (10^{-5} mbar) at room temperature.

X-ray absorption spectroscopy (XAS). X-ray absorption spectra at the Cu and Ga K-edge were measured at the SuperXAS beamline at the Swiss Light Source (SLS). The SLS is operating in top-up mode at a 2.4 GeV electron energy and a current of 400 mA. The incident photon beam provided by a 2.9 T super bend magnet source was selected by a Si(111) quick-EXAFS monochromator[5] and the rejection of higher harmonics and focusing were achieved by a silicon (for Cu K-edge) or a rhodium-coated (for Ga K-edge) collimating mirror at 2.5 mrad. During the measurements the monochromator was rotating with 10 Hz frequency and X-ray absorption spectra were collected in transmission mode using ionization chambers specially developed for quick data collection with 1 MHz frequency.[5] The resulting spectra were averaged over 5 min. Calibration of the monochromator energy position was performed by setting the inflection point of a Cu foil spectrum recorded simultaneously with the sample to 8979 eV for Cu. For the Ga K-edge, calibration was performed via a Pt foil by setting the inflection point at 11564 eV.

In a typical in situ experiment, about 10-20 mg of the powder sample was packed into a 3 mm thick quartz capillary (0.1 mm wall thickness), which was connected with a pressurizable gas flow system. The catalyst was reduced under a H_2/N_2 mixture (15%, 1 bar) at 300 °C for 60 min, and then cooled down to reaction temperature (230 °C). The reduction gas was flushed with N_2 for 15 min and then changed to the reaction gas mixture (CO_2:H_2:N_2 = 1:3:1, 5 ml min^{-1}). Under reaction gas, the set-up was pressurized to 5 bar using a back-pressure regulator by recording the spectra every 15 minutes for one hour or until no changes in the spectra occurred. The spectra were background-corrected and normalized using the Demeter software package. Ex situ samples were pressed in pellets with optimized thickness for transmission detection and placed in aluminized plastic bags (Polyaniline (14 μm), polyethylene (15 μm), Al (12 μm), polyethylene (75 μm)) from Gruber-Folien GmbH & Co. KG using an impulse sealer inside an argon filled glovebox to avoid air contamination. References (Cu, Pt and Ga_2O_3) were mixed with cellulose (in case of Ga_2O_3), pressed into wafers and sealed in kapton tape.

NMR. Solid-state NMR experiments on ^{1}H and ^{13}C were recorded on a Bruker 400 MHz AVANCE III HD spectrometer with a 4 mm MAS triple resonance probe operating in double resonance mode with a magic angle spinning frequency of 10 kHz. The chemical shift scale was calibrated using adamantane as an external secondary reference. Ramped cross polarization (^{1}H-^{13}C) was used for experiments with ^{1}H excitation frequency at 100 kHz. The contact time was 2 ms for 1D experiments and for ^{1}H-^{13}C HETCOR experiments. Additionally, for ^{1}H-^{13}C HETCOR experiment, DUMBO homonuclear (^{1}H-^{1}H) decoupling was used during t_1. The static magnetic field was externally referenced by setting the ^{13}C higher frequency peak of adamantane to 38.4 ppm. The ^{1}H excitation and decoupling radiofrequency (rf) fields were set as 100 kHz. CP conditions were optimized to fulfill the Hartmann-Hahn condition under magic-angle spinning with minor adjustments to reach optimal experimental CP efficiency. All samples were loaded in an argon filled glovebox.

Catalytic testing in CO_2 hydrogenation. CO_2 hydrogenation reactions were conducted in a fixed-bed tubular reactor (9.1 mm ID) in down-flow configuration (PID Eng&Tech). In a typical experiment 250 mg of catalyst powder oxidized in air was mixed with 5.0 g of SiC and loaded in the reactor under ambient conditions. First, the catalyst was reduced for 1 hour under a flow of 15% H_2/N_2 (50 ml min^{-1}) at 300 °C and atmospheric pressure. After cooling down to 230 °C, the reactor was pressurized to 25 bar with a flow of CO_2:H_2:N_2 (1:3:1, 50 ml min) for 30 min. The reactor was then set to measurement conditions (230 °C, 25 bar) and the gas phase was analyzed via online gas chromatography (Agilent 7890B) equipped with an FID for CH_3OH, CH_3OCH_3 and TCD for N_2, CO_2, CO and CH_4. Different contact times were probed by changing the gas flow rate from 100 mL (STP) min^{-1} to as low as 6 mL (STP) min^{-1}. Finally, activity data was collected at the initial flow rate of 100 mL min^{-1} to check for potential deactivation of the catalyst. The reaction rates, conversions and selectivities were calculated using the following set of equations (eq S2-S5):

$$F_{out}\left[mol\ h^{-1}\right] = \frac{F_{in} \cdot c_{N_2,in}}{c_{N_2,out}} \qquad \text{(S2)}$$

$$r_{Cu,x}[mol_x\ h^{-1}\ g_{Cu}^{-1}] = \frac{F_{out} \cdot c_{x,out}}{m_{cat} \cdot w_{Cu}} \quad \text{(S3)}$$

$$S_x = \frac{F_{x,out}}{\sum_{i=1}^{n} F_{i,out}} \quad \text{(S4)}$$

$$X_{CO_2} = \frac{\sum_{i=1}^{n} F_{i,out}}{F_{CO_2,in}} \quad \text{(S5)}$$

where F_{in} is the total gas inlet flowrate [mol h^{-1}], F_{out} is the total gas outlet flow rate [mol h^{-1}], $c_{x,in}$ is the inlet gas fraction of species x, $r_{Cu,x}$ is the formation rates of species x per gram copper $[g_x\ h^{-1}\ g_{Cu}^{-1}]$, m_{cat} is the mass of catalyst in the reactor $[g_{cat}]$, w_{Cu} the weight loading of copper $[wt_{Cu}\%,]$, S_x the product based selectivity of product x, $F_{i,out}$ the flowrates of the products, and X_{CO_2} the conversion of CO_2. Intrinsic formation rates (with respect to the contact time) and selectivity (with respect to CO_2 conversion) are obtained by using a second/third order polynomial fit on the experimental data. This function has no physical meaning and is merely used to allow estimating the initial rates and selectivity.

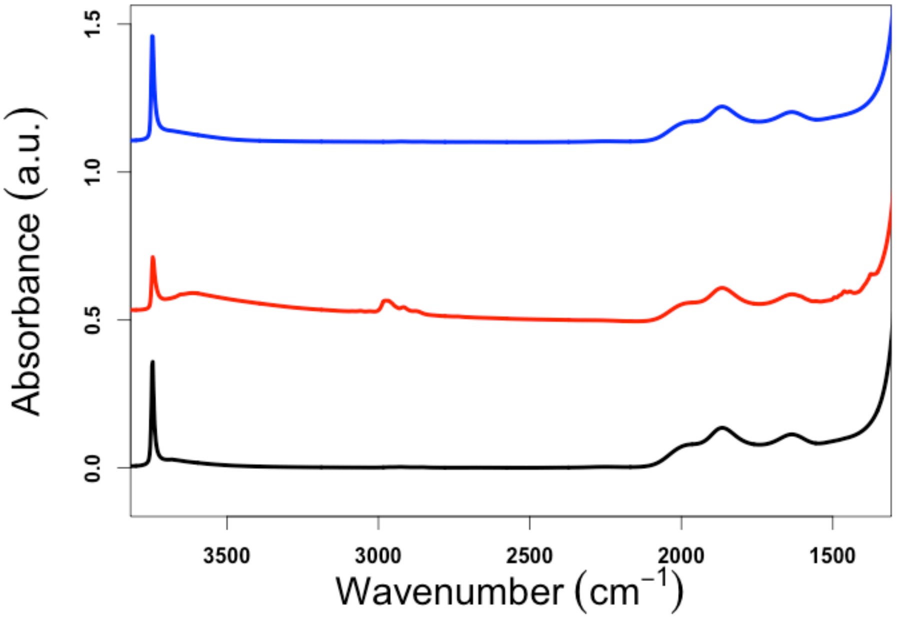

Figure S1. IR spectra of grafting and reduction of $[CuO^tBu]_4$ on **Ga^{III}@SiO$_2$** for forming **Cu-Ga/SiO$_2$** with **Ga^{III}@SiO$_2$** in black, the grafted material in red and **Cu-Ga/SiO$_2$** in blue.

Table S1. Physicochemical properties of copper based catalysts.

Entry	Catalyst	M loading[a)] [wt%]	Cu loading[a)] [wt%]	Specific surface area[b)] [m^2 g^{-1}]	Cu surface area based on N_2O[c)] [μmol g_{cat}^{-1}]	Cu surface area based on H_2[d)] [μmol g_{cat}^{-1}]	Cu particle size distribution[e)] [nm]
1	**Cu-Ga/SiO_2**	1.62	3.88	207	55/45	60	3.9 ± 1.0/ 4.9 ± 1.6
2 [ref. 3]	**Cu/SiO2**	N/A	4.19	197	50/34	N/A	2.9 ± 1.3/ 2.8 ± 1.3
3 [ref. 3]	**Cu/$Zr_{0.9}$@SiO2**	0.96	4.19	206	50/50	NA/	2.6 ± 1.3/ 2.7 ± 1.3

a) Determined by ICP-OES; b) determined from N_2 physisorption applying the BET theory; c) determined from N_2O chemisorption on fresh/spent catalyst; d) Measured on fresh catalyst, assuming a 1:2 stoichiometry between H_2 and Cu surface sites reduced with H_2 after exposure to air; e) determined by TEM of fresh/spent catalyst.

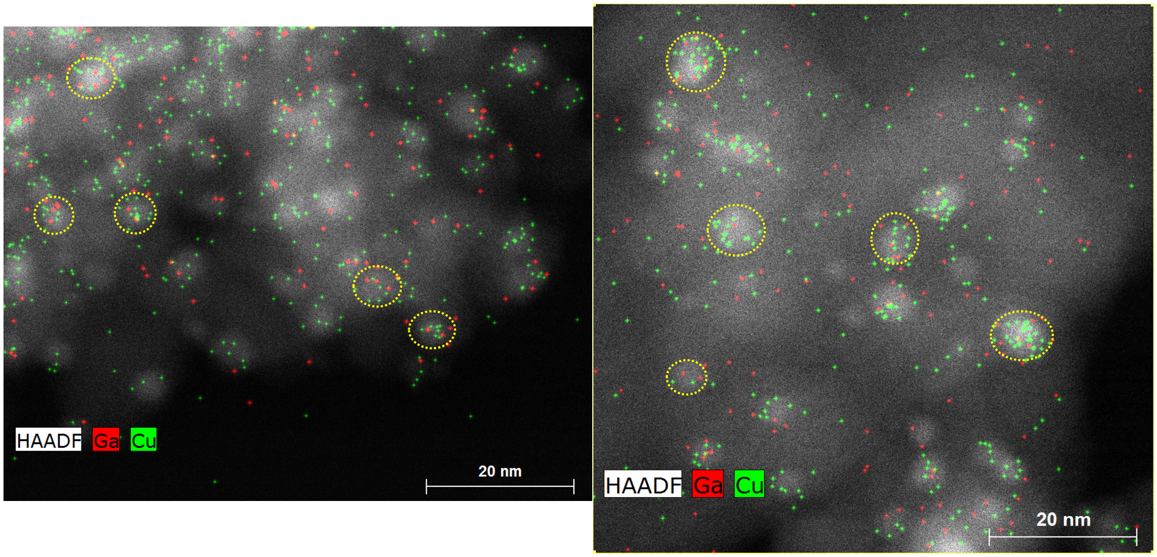

Figure S2. EDX mapping of **Cu-Ga/SiO_2** without exposure to air with the location of selected $CuGa_x$ nanoparticles indicated with yellow circles.

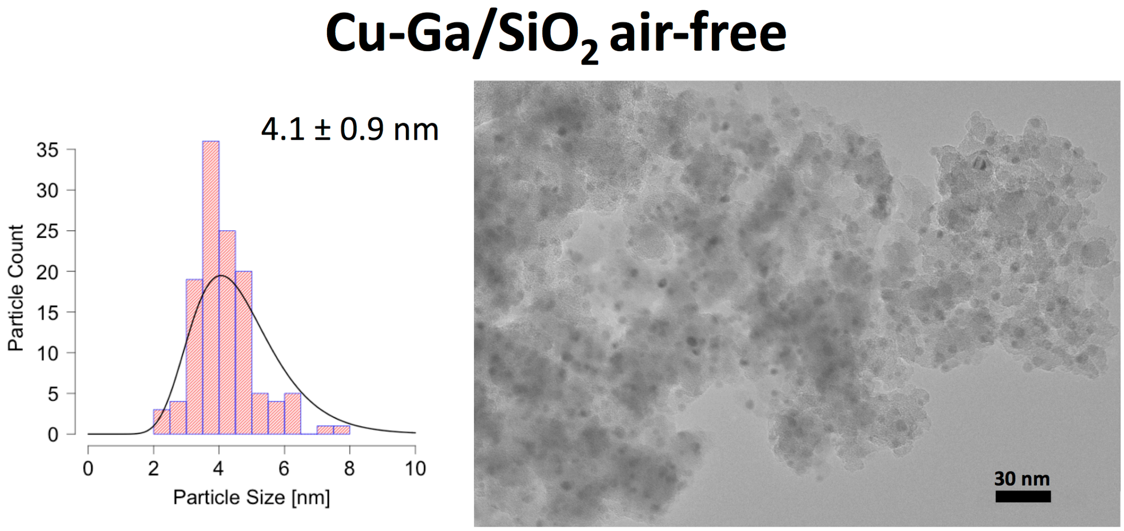

Figure S3. Particle size distribution and TEM imaging of **Cu-Ga/SiO$_2$** without exposure to air.

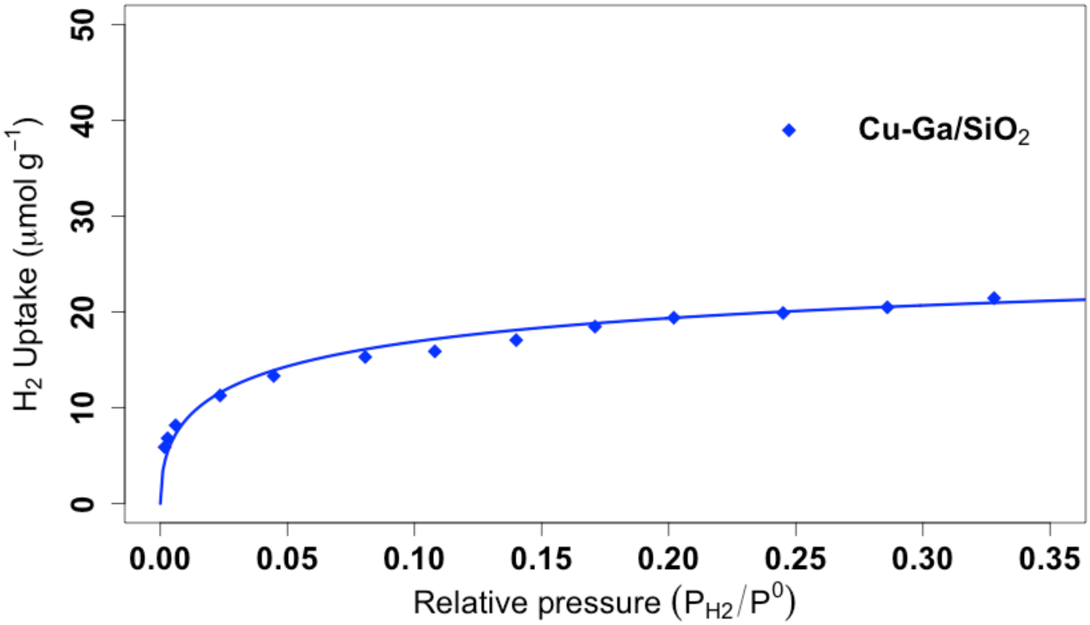

Figure S4. H_2 chemisorption isotherms recorded at 313 K and fitted curves using a Langmuir isotherm model for **Cu-Ga/SiO$_2$**.

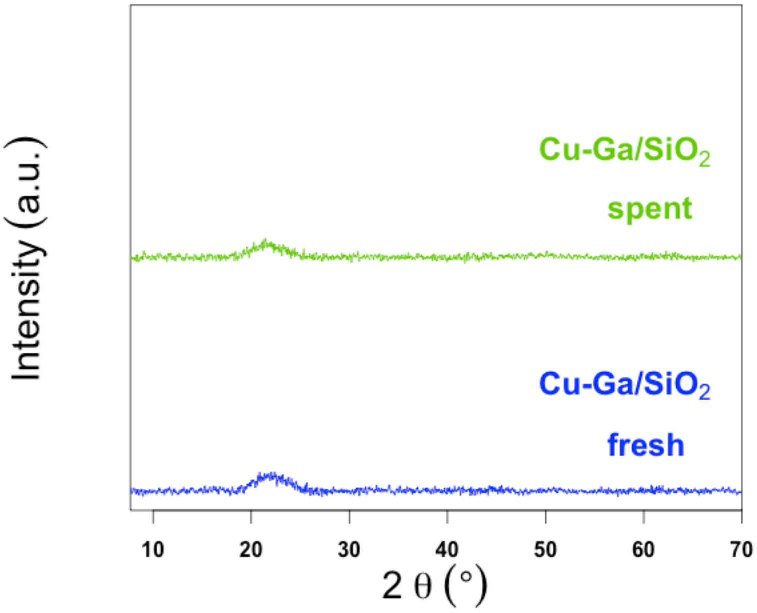

Figure S5. XRD pattern of fresh and spent **Cu-Ga/SiO_2**.

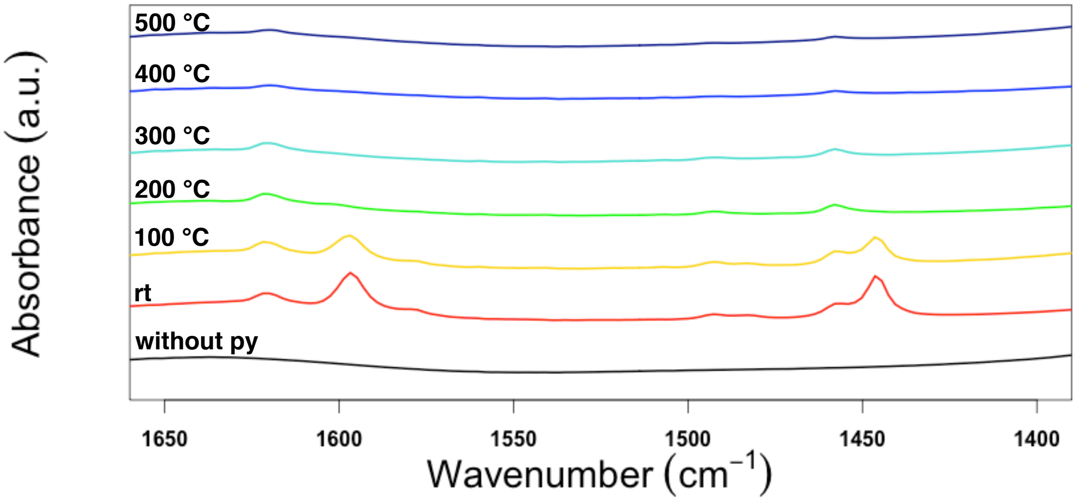

Figure S6. Pyridine adsorption experiment on **Cu-Ga/SiO_2** monitored by transmission IR after evacuation under high vacuum at different temperatures for at least 15 min.

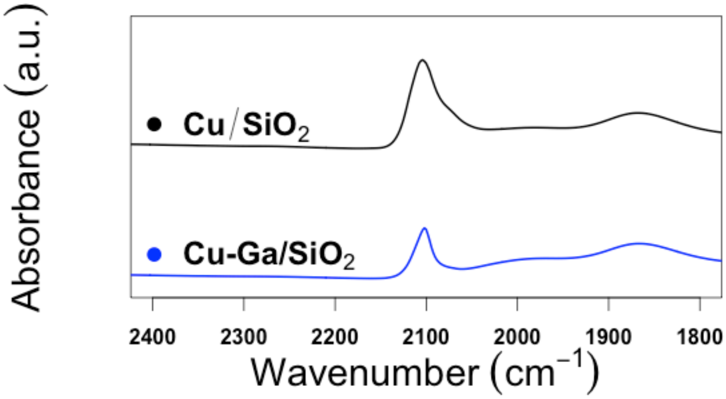

Figure S7. CO adsorption (90 mbar) on **Cu/SiO$_2$** and **Cu-Ga/SiO$_2$** monitored by transmission IR.

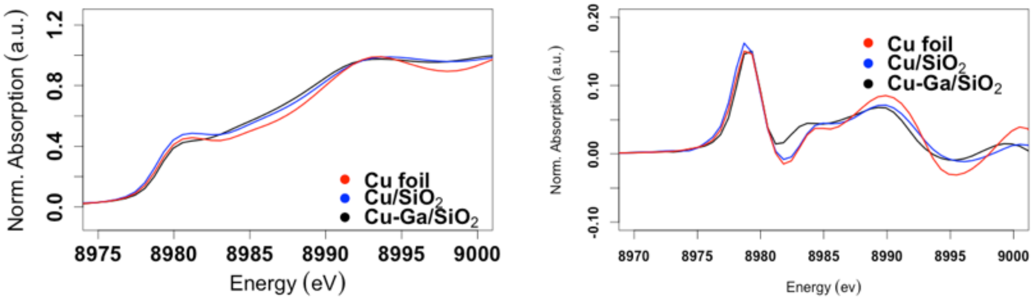

Figure S8. XANES spectrum (left) and its first derivative (right) at the copper K-edge for **Cu-Ga/SiO$_2$** and reference samples

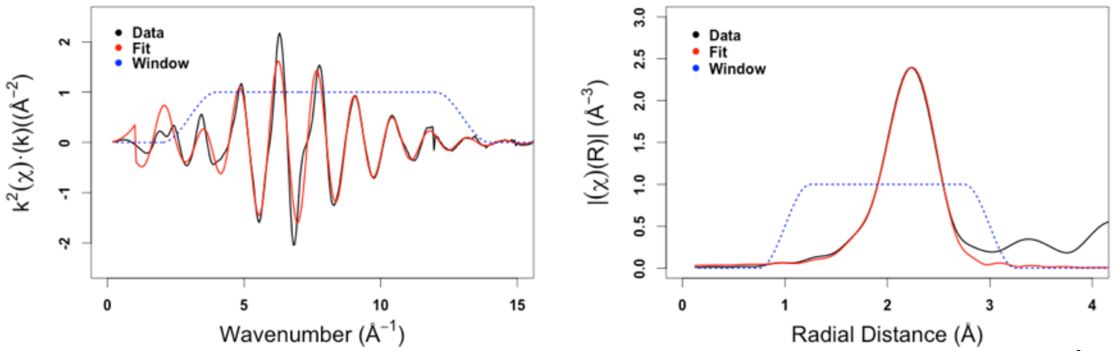

Figure S9. EXAFS data and fits in k-space (left) and R-space (right) of **Cu-Ga/SiO$_2$** at the Cu K-edge. $S0^2$ = 0.89; ΔE_0 = 3.8(7) eV; k range 3-13 $Å^{-1}$; R range 1-3 Å; K weight 2.

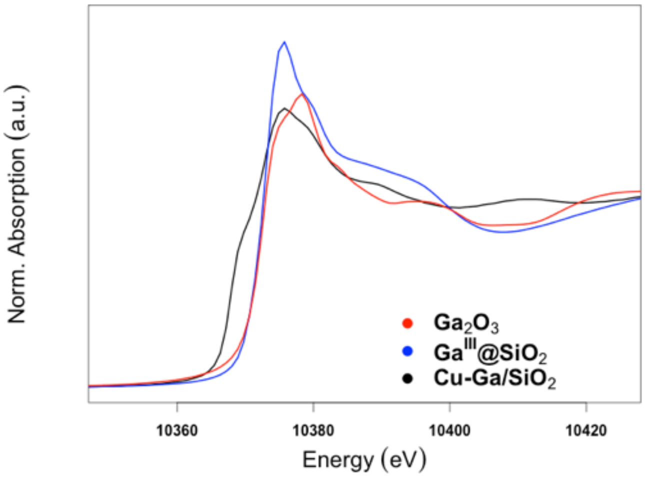

Figure S10. XANES spectrum at the gallium K-edge for **$Cu\text{-}Ga/SiO_2$** and reference samples.

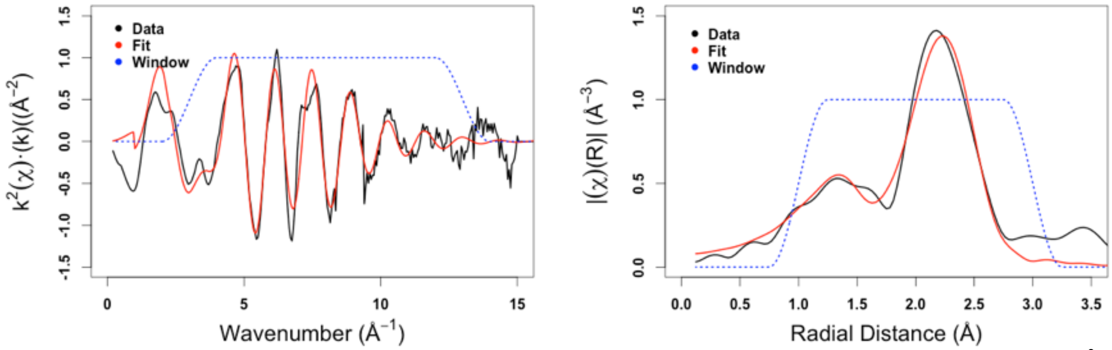

Figure S11. EXAFS data and fits in k-space (left) and R-space (right) of **$Cu\text{-}Ga/SiO_2$** at the Ga K-edge. $S0^2$ = 0.83; ΔE_0 = 3(2) eV; k range 3-13 $\AA^{-1}$; R range 1-3 Å; K weight 2.

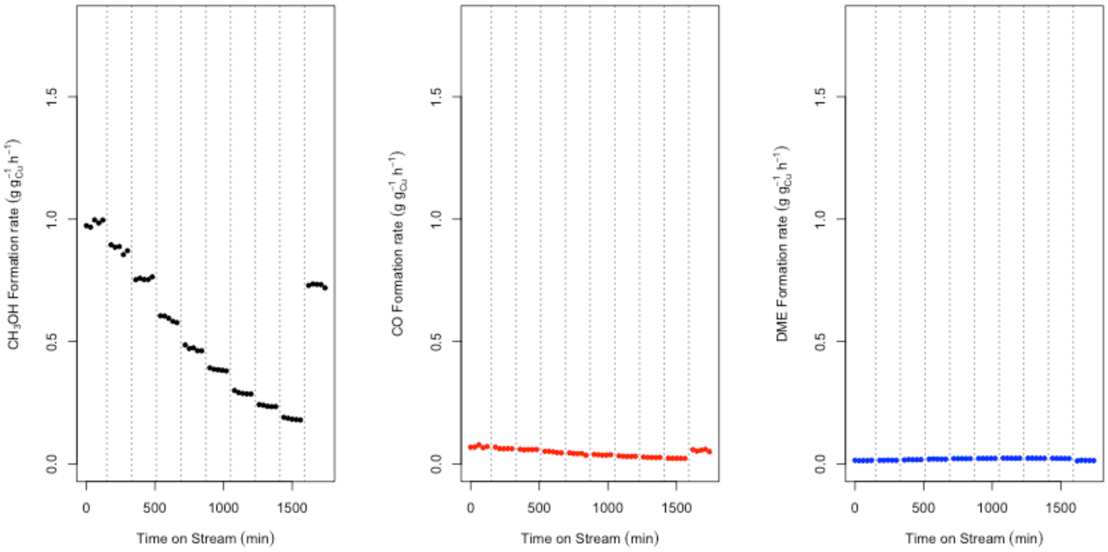

Figure S12. Formation rates during CO_2 hydrogenation of **Cu-Ga/SiO$_2$** in a flow reactor at 230 °C and 25 bars with 250 mg of catalyst for CH_3OH (left), DME (right) and CO (right) plotted with respect to time on stream CO_2:H_2:N_2 (1:3:1) with decreasing flowrates from 100, 75, 50, 30, 20, 15, 10, 8, 6 and 100 ml/min (separated by dotted lines).

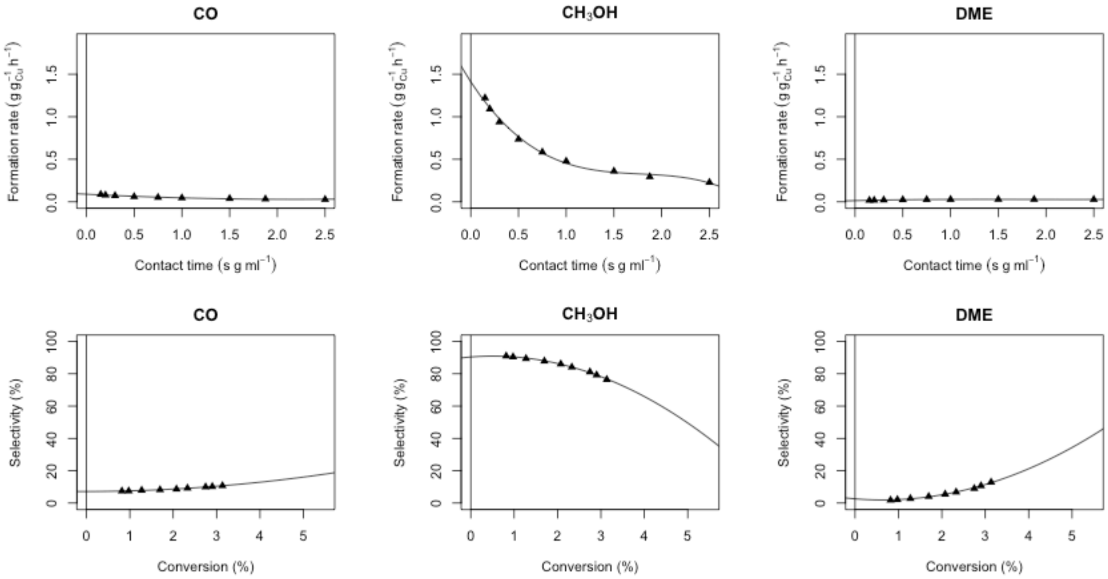

Figure S13. Catalytic data and fit of the formation rate vs. contact time and selectivity vs. conversion to obtain intrinsic formation rates and selectivity, respectively for **Cu-Ga/SiO$_2$.**

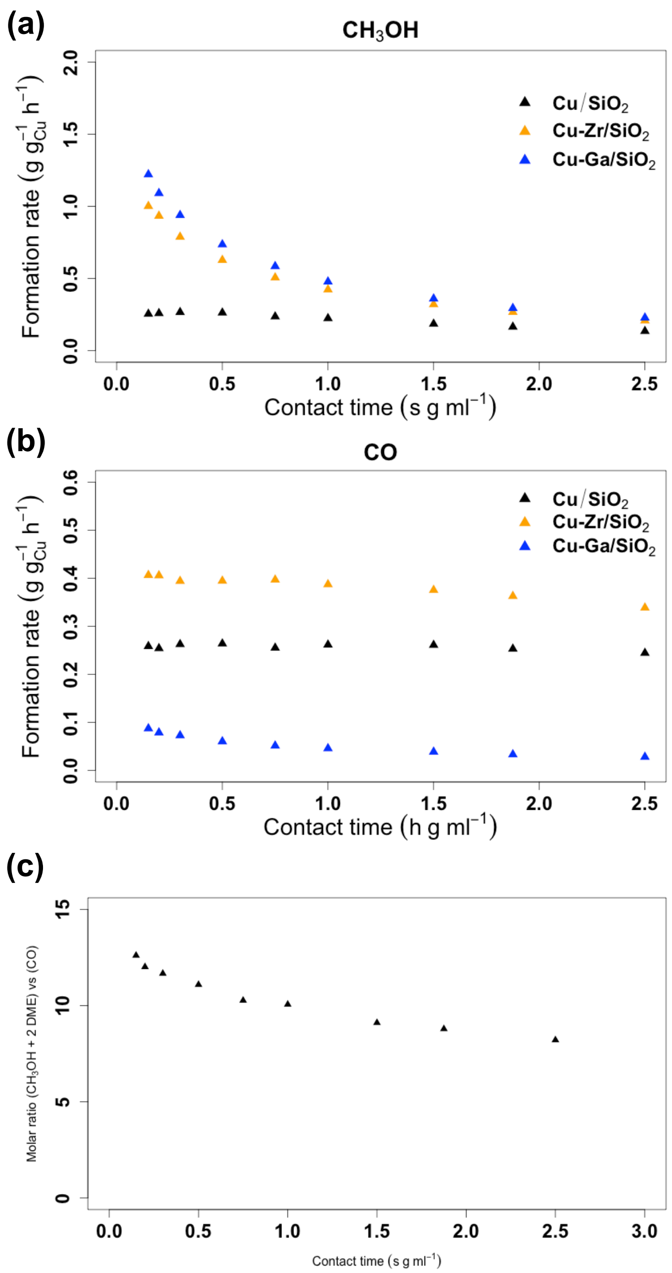

Figure S14. Formation rate with respect to contact time of for **Cu/SiO₂**, **Cu-Zr/SiO₂** and **Cu-Ga/SiO₂** for (a) CH_3OH and (b) CO. (c) Molar ratio of overall CH_3OH (CH_3OH + 2 DME) vs CO formation rate with respect to the contact time.

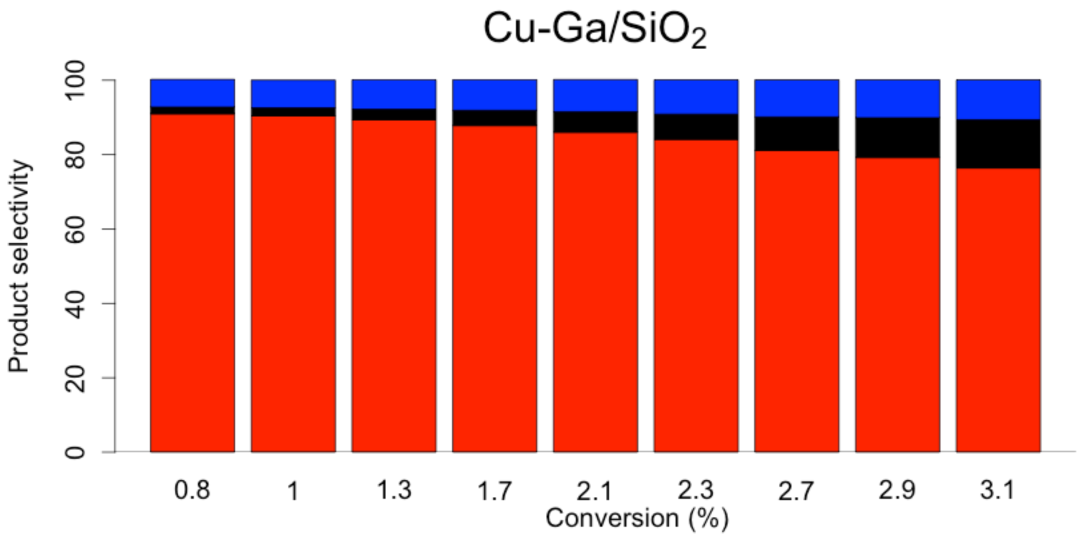

Figure S15. Conversion vs. selectivity of **Cu-Ga/SiO$_2$** at different conversions for CO (blue), DME (black) and CH_3OH (red).

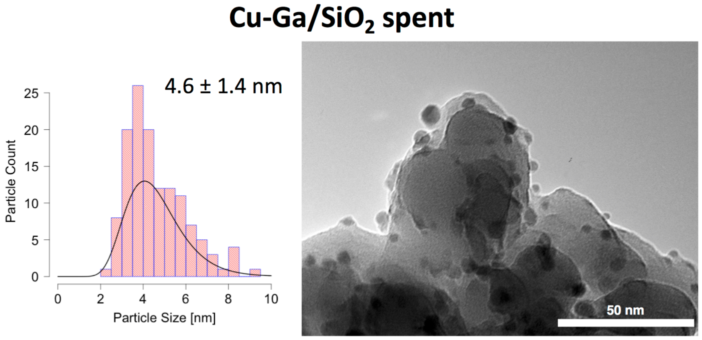

Figure S16. Particle size distribution and TEM imaging of **Cu-Ga/SiO$_2$** after catalysis.

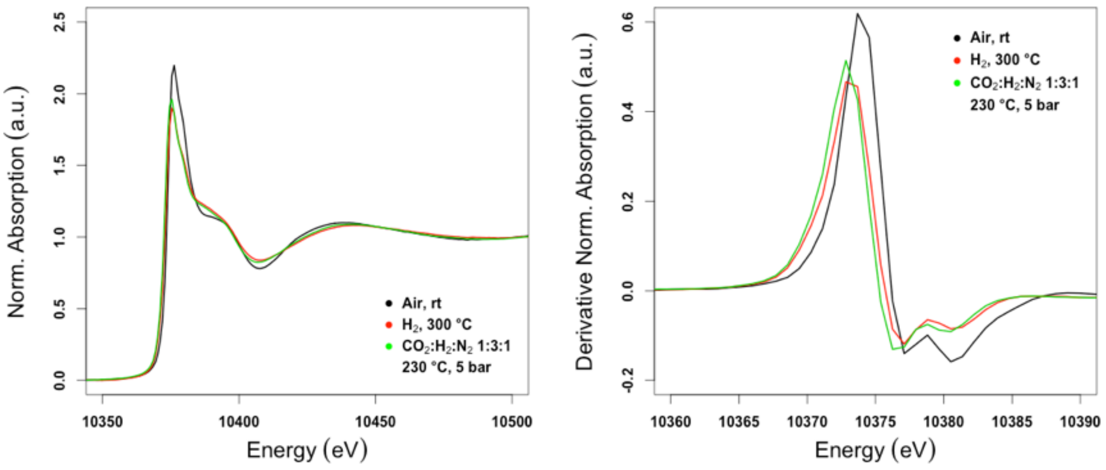

Figure S17. In situ XANES spectrum (left) and its first derivative (right) at the gallium K-edge for **Ga^{III}@SiO$_2$** under air at room temperature, reduced at 300 °C under H_2 and under reaction conditions at 5 bar with CO_2:H_2:N_2 (1:3:1) at 230 °C.

Table S2. EXAFS fits parameters of Ga@SiO_2 at the Ga K-edge.

Condition	Neighbor, N[a]	r [Å][b]	σ^2 [Å^2][c]
Air, rt	O, 5 ± 1	1.84 ± 0.02	0.005 ± 0.003
	O, 1.5 ± 0.6	2.03 ± 0.06	0.005 ± 0.003
H2, 300 °C	O, 3.0 ± 0.7	1.81 ± 0.02	0.002 ± 0.002
	O, 2.0 ± 0.6	2.00 ± 0.03	0.002 ± 0.002
CO2:H2:N2 (1:3:1)	O, 2.3 ± 0.5	1.79 ± 0.02	0.001 ± 0.003
230 °C, 5 bar	O, 2.4 ± 0.5	1.98 ± 0.02	0.001 ± 0.003

[a] Number of specified neighbors. [b] Distance to corresponding neighbor. [c] Debye-Waller factor.

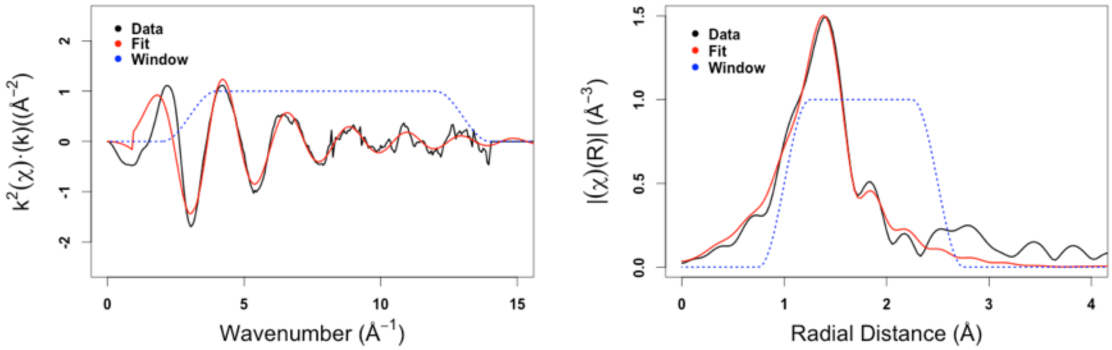

Figure S18. EXAFS data and fits in k-space (left) and R-space (right) of **Ga^{III}@SiO_2** at the Ga K-edge in air at room temperature. $S0^2$ = 0.83; ΔE_0 = 3(3) eV; k range 3-13 $Å^{-1}$; R range 1-2.5 Å; K weight 2.

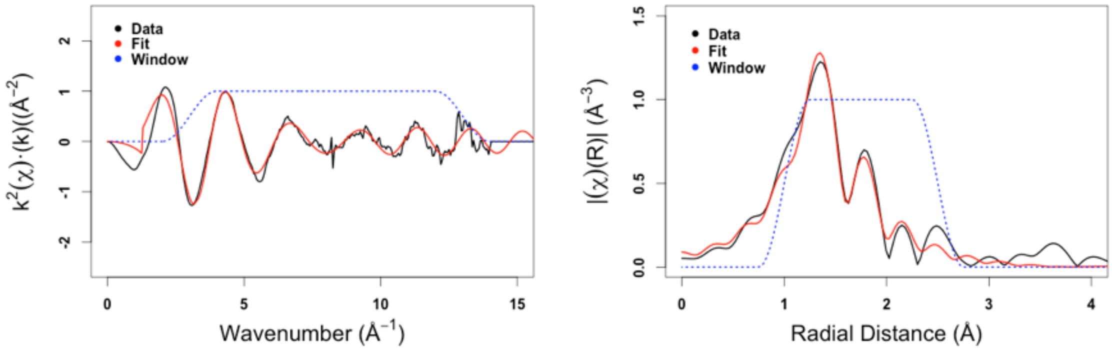

Figure S19. EXAFS data and fits in k-space (left) and R-space (right) of **Ga^{III}@SiO_2** at the Ga K-edge under a flow of H_2 at 300 °C. $S0^2$ = 0.83; ΔE_0 = 6(3) eV; k range 3-13 $Å^{-1}$; R range 1-2.5 Å; K weight 2.

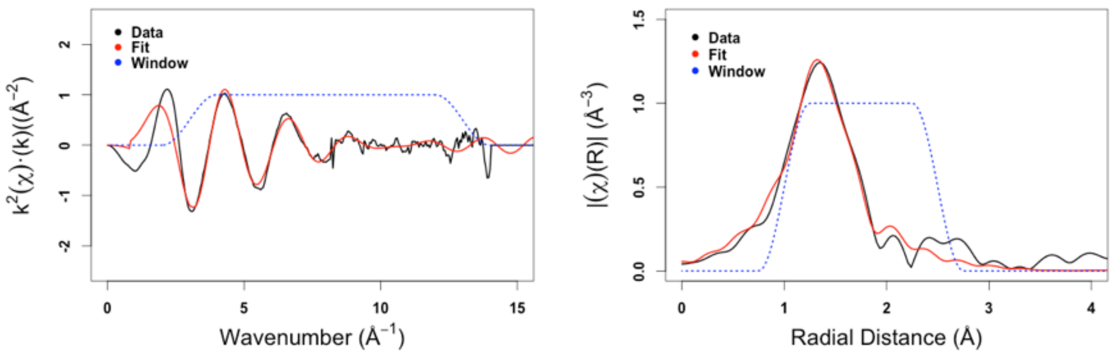

Figure S20. EXAFS data and fits in k-space (left) and R-space (right) of **Ga^{III}@SiO_2** at the Ga K-edge under a flow of CO_2:H_2:N_2 (1:3:1) at 230 °C and 5 bar. $S0^2 = 0.83$; $\Delta E_0 = 3(2)$ eV; k range 3-13 $Å^{-1}$; R range 1-2.5 Å; K weight 2.

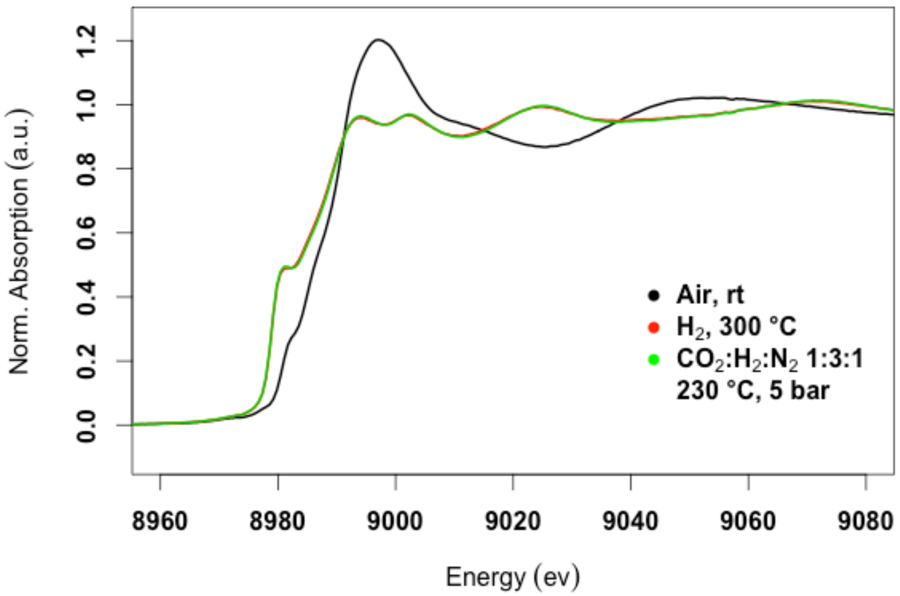

Figure S21. In situ XANES spectrum at the copper K-edge for **Cu-Ga/SiO_2** under air at room temperature, reduced at 300 °C under H_2 and under reaction conditions at 5 bar with CO_2:H_2:N_2 (1:3:1) at 230 °C.

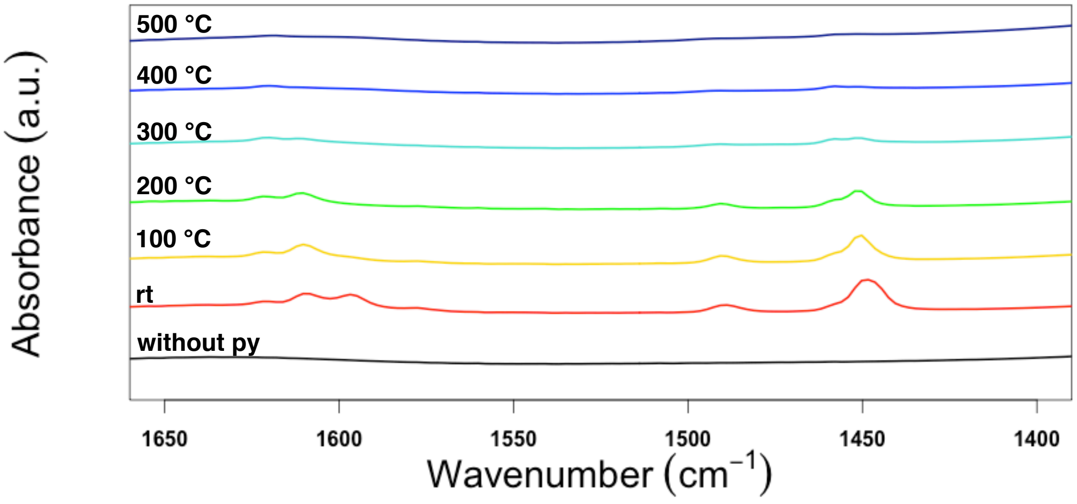

Figure S22. Pyridine adsorption experiment on **Cu-Ga/SiO$_2$** after exposure to air and reduction under H_2 at 300 °C monitored by transmission IR after evacuation under high vacuum at different temperatures for at least 15 min.

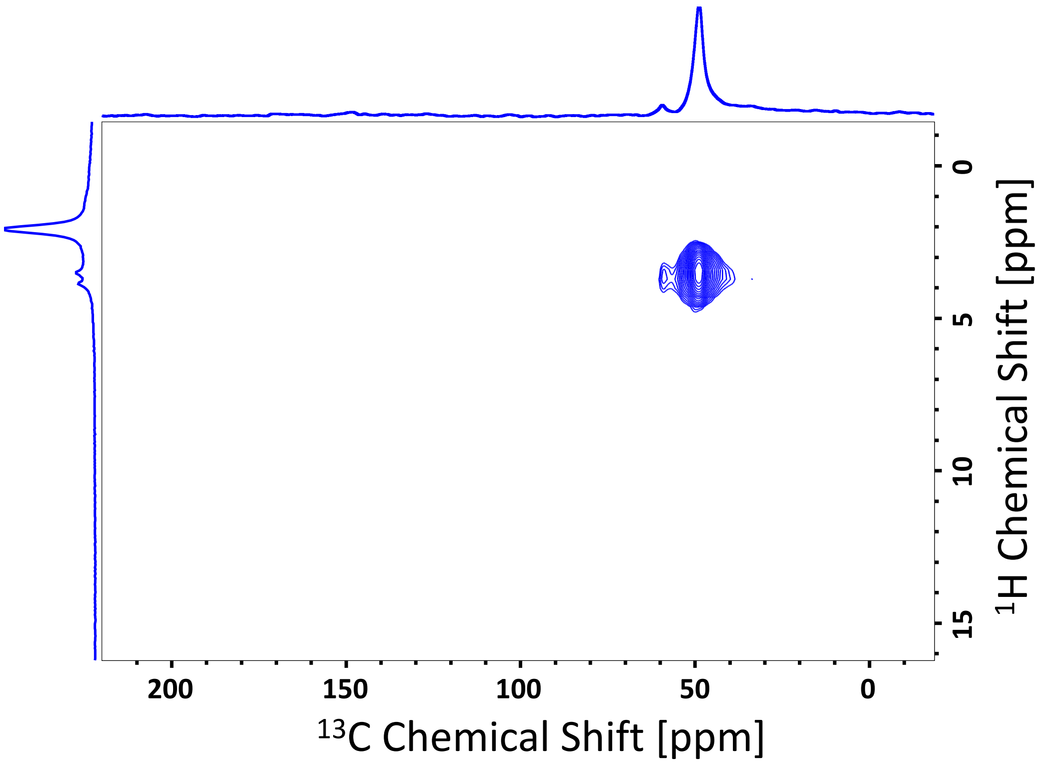

Figure S23. Ex-situ MAS-NMR ^{1}H -^{13}C HETCOR spectra of **Cu-Ga/SiO$_2$** reacted with CO_2:H_2 (1:3) at 5 bars for 12 hours and 230 °C.

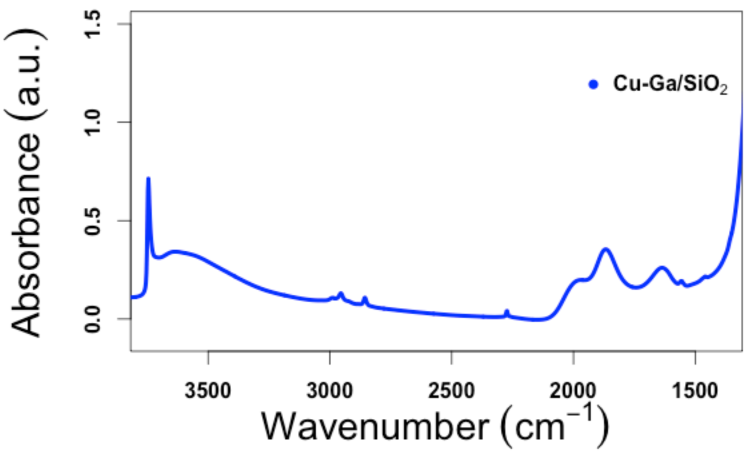

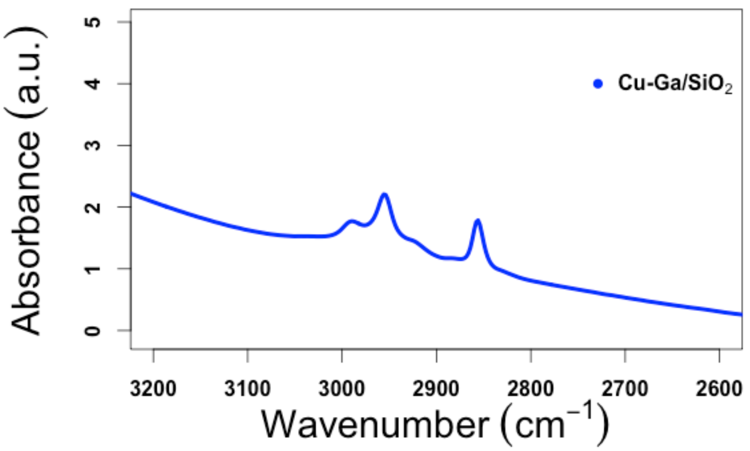

Figure S24. Ex-situ IR spectra of **Cu-Ga/SiO$_2$** reacted with CO_2:H_2 (1:3) at 5 bars for 12 hours and 230 °C.

References

1. A. Roussey, P. Gentile, D. Lafond, E. Martinez, V. Jousseaume, C. Thieuleux and C. Coperet, *J. Mater. Chem. C*, 2013, **1**, 1583-1587.
2. K. Searles, G. Siddiqi, O. V. Safonova and C. Copéret, *Chem. Sci.*, 2017, **8**, 2661-2666.
3. E. Lam, K. Larmier, P. Wolf, S. Tada, O. V. Safonova and C. Copéret, *J. Am. Chem. Soc.*, 2018, **140**, 10530-10535.
4. K. Larmier, S. Tada, A. Comas-Vives and C. Copéret, *J. Phys. Chem. Lett.*, 2016, **7**, 3259-3263.
5. O. Muller, M. Nachtegaal, J. Just, D. Lutzenkirchen-Hecht and R. Frahm, *J. Synchrotron Radiat.*, 2016, **23**, 260-266.
